# Integrin Activation States and Eosinophil Recruitment in Asthma

**DOI:** 10.3389/fphar.2013.00033

**Published:** 2013-04-01

**Authors:** Mats W. Johansson, Deane F. Mosher

**Affiliations:** ^1^Department of Biomolecular Chemistry, University of WisconsinMadison, WI, USA; ^2^Department of Medicine, University of WisconsinMadison, WI, USA

**Keywords:** eosinophils, integrins, adhesion, asthma, inflammation

## Abstract

Eosinophil arrest and recruitment to the airway in asthma are mediated, at least in part, by integrins. Eosinophils express α_4_β_1_, α_6_β_1_, α_L_β_2_, α_M_β_2_, α_X_β_2_, α_D_β_2_, and α_4_β_7_ integrins, which interact with counter-receptors on other cells or ligands in the extracellular matrix. Whether a given integrin-ligand pair mediates cell adhesion and migration depends on the activation state of the integrin. Integrins exist in an inactive bent, an intermediate-activity extended closed, and a high-activity extended open conformation. Integrin activation states can be monitored by conformation-specific monoclonal antibodies (mAbs). Studies in mice indicate that both β_1_ and β_2_ integrins mediate eosinophil recruitment to the lung. *In vitro* studies indicate that α_4_β_1_ and α_M_β_2_ are the principal integrins mediating eosinophil adhesion, including to vascular cell adhesion molecule-1 and the novel α_M_β_2_ ligand periostin. *In vivo*, blood eosinophils have intermediate-activity β_1_ integrins, as judged by mAb N29, apparently resulting from eosinophil binding of P-selectin on the surface of activated platelets, and have a proportion of their β_2_ integrins in the intermediate conformation, as judged by mAb KIM-127, apparently due to exposure to low concentrations of interleukin-5 (IL-5). Airway eosinophils recovered by bronchoalveolar lavage (BAL) after segmental antigen challenge have high-activity β_1_ integrins and high-activity α_M_β_2_ that does not require IL-5. Here we review information on how the activation states of eosinophil β_1_ and β_2_ integrins correlate with measurements of eosinophil recruitment and pulmonary function in asthma. Blood eosinophil N29 reactivity is associated with decreased lung function under various circumstances in non-severe asthma and KIM-127 with BAL eosinophil numbers, indicating that intermediate-activity α_4_β_1_ and α_M_β_2_ of blood eosinophils are important for eosinophil arrest and consequently for recruitment and aspects of asthma.

## Introduction

Eosinophilic airway inflammation is frequent pattern in different phenotypes or endotypes of asthma (Busse and Lemanske, 2001; Scott and Wardlaw, 2006; Simpson et al., 2006; Anderson, 2008; Haldar et al., 2008; Hogan et al., 2008; Blanchard and Rothenberg, 2009; Woodruff et al., 2009; Hastie et al., 2010; Lee et al., 2010; Moore et al., 2010; Kita, 2011; Lotvall et al., 2011; Siroux et al., 2011; Wang et al., 2011; Agache et al., 2012; Wenzel, 2012; Rosenberg et al., 2013). Airway eosinophilia is associated with exacerbations (Haldar et al., 2009; Nair et al., 2009; Busse et al., 2010; Pavord et al., 2012; Robinson, 2013) and has been suggested to play a role in airway remodeling (Flood-Page et al., 2003; Kay et al., 2004; Wills-Karp and Karp, 2004; Busse et al., 2010; Robinson, 2013). Arrest of eosinophils in vessels and their extravasation into the airway wall and through the bronchial tissue and epithelium to the airway lumen are mediated in part by the integrin family of cell adhesion receptors (Banerjee et al., 2007, 2009; Rosenberg et al., 2007; Barthel et al., 2008).

**Figure 1:**
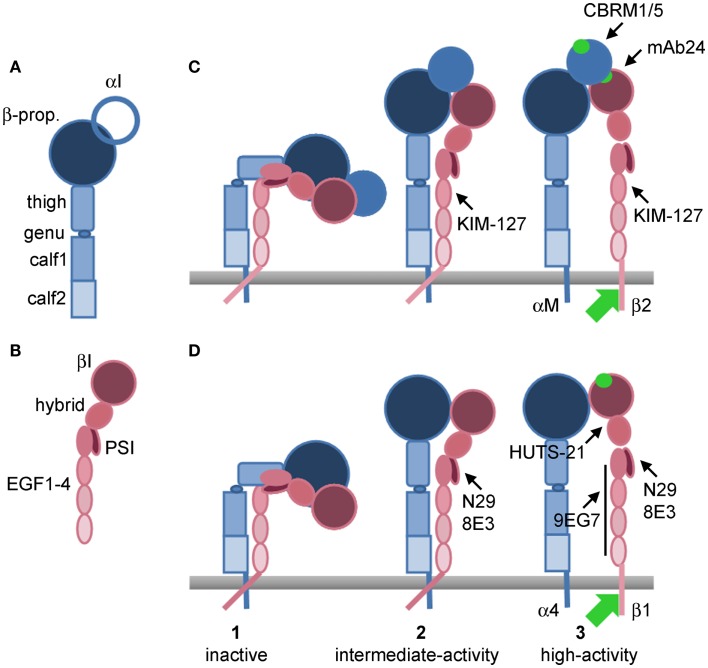
**Models of integrin conformations with epitopes for activation-sensitive mAbs**. **(A)** Domains of an integrin α subunit. **(B)** Domains of an integrin β subunit. **(C)** Conformational changes during activation of α_M_β_2_ that uncover epitopes for anti-β_2_ KIM-127 and mAb24, and anti-α_M_ CBRM1/5. **(D)** Conformational changes during activation of α_4_β_1_ that uncover epitopes for anti-β_1_ N29, 8E3, HUTS-21, and 9EG7. (1) Inactive, bent conformations; (2) intermediate-activity, extended, closed conformations; (3) high-activity, extended, open conformations. In **(C)** conformation 1 of α_M_β_2_ is presumably KIM-127−/mAb24−/CBRM1/5−, conformation 2 KIM-127+/mAb24−/CBRM1/5−, and conformation 3 KIM-127+/mAb24+/CBRM1/5+. In **(D)** conformation 1 of α_4_β_1_ is presumably N29−/8E3−/HUTS-21−/9EG7−, conformation 2 N29+/8E3+/HUTS-21−/9EG7−, and conformation 3 N29+/8E3+/HUTS-21+/9EG7+. Green circle, ligand-binding site in αI domain in **(C)** or βI domain in **(D)**, or binding site in βI domain for activated αI domain in **(C)**. Green arrow, cytoplasmic proteins, including talin and kindlins, that bind the β integrin subunit tail and mediate activation. β-prop., β-propeller domain; EGF, integrin epidermal growth factor-like domain; PSI, plexin-semaphorin-integrin domain. Based on (Luo and Springer, 2006; Luo et al., 2007; Barthel et al., 2008; Evans et al., 2009; Hogg et al., 2011).

Integrins are heterodimers of α and β subunits (Hynes, 1987; Ruoslahti, 1991). Each integrin interacts or potentially interacts with counter-receptors on other cells or ligands deposited as part of the extracellular matrix (ECM) (Humphries et al., 2006). Whether a given pair of integrin and counter-receptor/ECM ligand participates in cell adhesion and migration depends on the cell-surface density of the integrin, the density of the ligand, and the activation state of the integrin (Huttenlocher et al., 1996; Palecek et al., 1997; Askari et al., 2009). Integrins exist in three major conformations, including an inactive or low-activity, bent conformation; an intermediate-activity, extended, “closed” conformation with a partially occluded ligand-binding “head piece”; and a high-activity, extended, “open” conformation with a more open “head piece,” swung-out hybrid domain, and separation of the “legs” of the two subunits (Figure 1) (Luo and Springer, 2006; Luo et al., 2007; Evans et al., 2009; Hogg et al., 2011; Margadant et al., 2011). The activation states of integrins can be monitored by conformation-specific monoclonal antibodies (mAbs) (Table 1; Figure 1) (Humphries, 2004; Byron et al., 2009). Integrins are activated through so-called “inside-out” signaling by signals triggered through other receptors, including G-protein coupled receptors (GPCRs) for chemokines, and mediated by cytoplasmic factors including talin and kindlins that bind the cytoplasmic tail of integrin β subunits (Evans et al., 2009; Harburger and Calderwood, 2009; Shattil et al., 2010; Hogg et al., 2011; Margadant et al., 2011). Engagement of integrins by ligands, in turn, can activate cytoplasmic signaling pathways, “outside-in” signaling (Harburger and Calderwood, 2009; Margadant et al., 2011).

**Table 1:** **Some activation-sensitive anti-integrin antibodies**.

Antibody	Integrin subunit	Epitope location	Recognized conformation(s)	Reference
N29	β_1_	PSI domain	Intermediate- and high-activity	Wilkins et al. (1996), Ni et al. (1998), Mould et al. (2005), Barthel et al. (2008), Byron et al. (2009)
8E3	β_1_	PSI domain	Intermediate- and high-activity	Coe et al. (2001), Mould et al. (2005), Barthel et al. (2008), Byron et al. (2009)
HUTS-21	β_1_	Hybrid domain	High-activity	Luque et al. (1996), Barthel et al. (2008)
9EG7	β_1_	EGF-like domains	High-activity	Lenter et al. (1993), Bazzoni et al. (1995), Barthel et al. (2008), Byron et al. (2009)
KIM-127	β_2_	2nd EGF-like domain	Intermediate- and high-activity	Robinson et al. (1992), Beglova et al. (2002), Byron et al. (2009), Evans et al. (2009)
mAb24	β_2_	I domain	High-activity	Dransfield and Hogg (1989), Leitinger and Hogg (2000), Lu et al. (2001), Byron et al. (2009), Evans et al. (2009)
CBRM1/5	α_M_	I domain	High-activity	Oxvig et al. (1999), Byron et al. (2009)

*EGF, epidermal growth factor; PSI, plexin-semaphorin-integrin*.

## Eosinophil Integrins

We briefly discuss the eosinophil integrin repertoire and their counter-receptors, ligands or potential ligands here, focusing on recent novel information. For a more extensive overview over eosinophil integrins and their ligands, including potential functions of the integrins that are not the focus here, we refer the reader to our previous review (Barthel et al., 2008). Eosinophils possess a unique repertoire of seven integrins, α_4_β_1_, α_6_β_1_, α_L_β_2_, α_M_β_2_, α_X_β_2_, α_D_β_2_, and α_4_β_7_ integrins (Barthel et al., 2008; Hogan et al., 2008). Eosinophil integrins have the potential to mediate adhesion and migration on vascular cell adhesion molecule-1 (VCAM-1) via α_4_β_1_, α_M_β_2_, α_X_β_2_, α_D_β_2_, and α_4_β_7_; intercellular adhesion molecule-1 (ICAM-1) via α_L_β_2_ and α_M_β_2;_ laminin via α_6_β_1_; fibrinogen/fibrin via α_M_β_2_ and α_X_β_2_; and vitronectin via α_M_β_2_ (Barthel et al., 2008). In our hands, purified human blood eosinophils, in the absence of added soluble stimulus, adhere *in vitro* specifically only to VCAM-1, primarily via α_4_β_1_. Eosinophils from some subjects and eosinophils under flow conditions also adhere to VCAM-1 via α_M_β_2_ (Barthel et al., 2006a,b). Purified airway eosinophils, recovered by bronchoalveolar lavage (BAL) 48 h after segmental lung antigen challenge (a model of allergic airway inflammation), or blood eosinophils stimulated with interleukin-5 (IL-5), adhere specifically to VCAM-1 via α_4_β_1_ and α_M_β_2_, and to ICAM-1, fibrinogen, and vitronectin via α_M_β_2_ (Barthel et al., 2006b). In addition, we recently discovered that blood eosinophils stimulated with IL-5, IL-3, or granulocyte macrophage-colony stimulating factor (GM-CSF) specifically adhere to and migrate on periostin via α_M_β_2_ (Johansson et al., 2013b). Periostin is an ECM protein upregulated by T helper cell type 2 (Th2) cytokines in bronchial epithelial cells and lung fibroblasts and is deposited in patients with asthma and atopic dermatitis, as well as in animal models of asthma and allergic skin inflammation (Yuyama et al., 2002; Takayama et al., 2006; Hayashi et al., 2007; Woodruff et al., 2007; Masuoka et al., 2012). Mice lacking periostin respond to lung antigen challenge with significantly decreased number of eosinophils in the lung and have reduced allergic skin inflammation (Blanchard et al., 2008; Bentley et al., 2012; Masuoka et al., 2012), thus implicating periostin as a ligand in eosinophil recruitment and retention in allergy and asthma. Further, studies of β_2_-deficient and conditionally α_4_-deficient mice indicate that both β_2_ and α_4_, presumably principally α_4_β_1_, integrins mediate trafficking of eosinophils to the lung in models of allergen-induced acute and chronic asthma (Banerjee et al., 2007, 2009).

Taken together, these studies in mice and men indicate that α_4_β_1_ and α_M_β_2_ are the major eosinophil integrins mediating cell adhesion, with α_4_β_1_ largely responsible for arrest of blood eosinophils on VCAM-1 in vessels of the asthmatic lung, with a more minor contribution by α_M_β_2_; whereas activated α_M_β_2_, by interacting with periostin and possibly other ligands, is involved in subsequent eosinophil recruitment to and persistence in the ECM of the bronchi in asthma.

## Activation States of Integrins on Blood and Airway Eosinophils

As purification of eosinophils leads to increased partial activation of β_1_ integrins, assessed by mAb N29 (Johansson and Mosher, 2011); we monitor activation states of integrins on blood and airway eosinophils by processing unfractionated blood or BAL cells for flow cytometry and analyzing eosinophils, which are gated to exclude other cells, including neutrophils, monocytes, lymphocytes, and NK cells, based on CD14 and CD16 staining and scatter (Johansson et al., 2006, 2008, 2012, 2013a; Johansson and Mosher, 2011).

On the average, eosinophils in blood express the N29 and 8E3 epitopes to some degree but have no or very low expression of the HUTS-21 and 9EG7 epitopes, indicating that their β_1_ integrins, including α_4_β_1_, are in the intermediate-, but not high-activity, conformation (Table 1; Figure 1) (Johansson et al., 2006, 2008, 2012, 2013a; Johansson and Mosher, 2011). However, N29 and 8E3 reactivities are variable among subjects, ranging from some subjects with essentially no N29 signal and thus inactive β_1_ integrins to some with low but detectable N29 signal (i.e., a fraction of β_1_ integrin molecules on each cell having the intermediate-activity conformation) to some with high N29 signal (i.e., presumably most molecules having the intermediate-activity conformation) (Johansson et al., 2008, 2012; Johansson and Mosher, 2011). As a group, subjects with asthma or subjects with non-severe asthma, but not subjects with severe asthma, have a higher N29 signal than normal donors (Johansson et al., 2012). In subjects with non-severe allergic asthma who have a dual response phenotype (i.e., they have a fall in forced expiratory volume in 1 s (FEV_1_) of ≥15% during the late-phase 3–8 h after whole-lung antigen challenge, in addition to the common initial early-phase fall within 15–30 min), N29 reactivity was increased 48 h after segmental lung antigen challenge (Johansson et al., 2008). After whole-lung antigen challenge itself, which is a more major insult and a model of asthma exacerbation (Gauvreau and Evans, 2007), N29 reactivity of circulating eosinophils decreases at 8 h and recovers at 48 h, indicating that cells with the highest proportion of activated α_4_β_1_ are the ones that extravasate. We have suggested that a similar phenomenon, i.e., that the eosinophils with the most activated α_4_β_1_ are efficiently removed, occurs continuously in severe asthma, compatible with the data that the N29 signal on circulating cells in severe asthma is not significantly higher than in normal donors (Johansson et al., 2012). Efficient removal in severe asthma may be due to greater lung endothelial VCAM-1 expression, as has been observed in bronchial biopsies of subjects with severe compared to non-severe asthma (Ramos-Barbon et al., 2010). Taken together, this information indicates that a proportion, variable among subjects, of the α_4_β_1_ molecules on blood eosinophils are in an intermediate conformation. This proportion is higher in asthma than in healthy donors, and can increase, e.g., upon segmental antigen challenge, but can also decrease when or if the most activated cells extravasate, such as after whole-lung challenge or presumably continuously in severe asthma.

Purified blood eosinophils are positive for N29 but not HUTS-21 and 9EG7, and adhere “constitutively” to VCAM-1 *in vitro* in an α_4_β_1_-dependent manner (Barthel et al., 2006a,b); compatible with a situation in which all or most of their α_4_β_1_ molecules are in the intermediate conformation. However, purified cells are not a completely accurate reflection of eosinophils *in vivo*, since, as indicated above, the N29 signal increases upon cell purification (Johansson and Mosher, 2011). Thus, as eosinophils from different subjects *in vivo* have variable N29 reactivity, it is reasonable to assume that the capacity of eosinophils to arrest on VCAM-1 *in vivo* also varies among subjects.

Regarding β_2_ integrins, eosinophils in blood (from subjects with non-severe asthma) have a low but detectable KIM-127 signal but very low reactivity with mAb24 and CBRM1/5, indicating that a fraction of their β_2_ integrins, including α_M_β_2_, is in the intermediate- (but not high-) activity conformation (Johansson et al., 2008, 2013a). In consistency with this, purified blood eosinophils do not react with CBRM1/5 and do not adhere *in vitro* to the α_M_β_2_ ligands ICAM-1, fibrinogen, vitronectin, and periostin, unless IL-5 is added to stimulate the cells (Barthel et al., 2006b; Johansson et al., 2013b). However, since, as mentioned, α_M_β_2_, in addition to α_4_β_1_, is involved in adhesion of purified blood eosinophils from some subjects to VCAM-1 and mediates arrest to VCAM-1 under flow (Barthel et al., 2006a), a proportion of α_M_β_2_ on blood eosinophils in the intermediate conformation is likely sufficient for α_M_β_2_ to participate, together with α_4_β_1_, in arrest to VCAM-1.

Eosinophils in BAL obtained after segmental antigen challenge have β_1_ integrins in the high-activity conformation, judged by their significantly increased reactivity with HUTS-21 and 9EG7 compared to blood eosinophils (Johansson et al., 2013a). α_M_β_2_ on such BAL eosinophils also is in a high-activity conformation, as assessed by the higher reactivity with mAb24 and CBRM1/5 than on blood eosinophils (Johansson et al., 2008, 2013a) and very high reactivity with KIM-127 (Johansson et al., 2013a). These flow cytometry data on eosinophils in BAL agree with the fact that purified airway eosinophils adhere to VCAM-1 to a higher degree than blood eosinophils in a process involving both α_4_β_1_ and α_M_β_2_, and adhere to ICAM-1, fibrinogen, and vitronectin in a α_M_β_2_-dependent manner (Barthel et al., 2006b).

## Activating Stimuli

Addition of soluble P-selectin to whole blood *in vitro* increases the N29 signal of blood eosinophils and their adhesion to VCAM-1 (Johansson and Mosher, 2011), thus enhancing the activation of α_4_β_1_. Added P-selectin does not enhance the N29 signal or adhesion of purified blood eosinophils to VCAM-1, in consistency with the fact that N29 is increased and thus α_4_β_1_ is activated during cell purification (Johansson and Mosher, 2011). We suspect that in the process of purification, contaminating platelets are activated, externalizing P-selectin and in turn activating eosinophils. In whole blood samples, eosinophil reactivity with N29 or 8E3 correlates with the amount of P-selectin associated with the eosinophil surface in non-severe asthma (Table 2) (Johansson and Mosher, 2011), and N29 does so also in a population of subjects with asthma of varying severity (Table 2) (Johansson et al., 2012). Further, eosinophil N29 correlates with platelet-surface P-selectin expression (Johansson et al., 2012). These correlations support a scenario in which P-selectin present on the surface of activated platelets is the *in vivo* stimulus that by binding to eosinophils, presumably via P-selectin glycoprotein ligand-1 (PSGL-1), the eosinophil counter-receptor for P-selectin (Symon et al., 1996), triggers an intracellular signaling pathway that results in conversion of inactive to intermediate-activity α_4_β_1_ and the stimulation of arrest on VCAM-1. As with the N29 signal, after whole-lung antigen challenge, eosinophil-bound P-selectin and eosinophil PSGL-1 decreased at 8 h and recovered at 48 h (Johansson et al., 2012), indicating that the cells with the highest PSGL-1 level and P-selectin binding, as well as highest degree of α_4_β_1_ activation, extravasate. This situation is compatible with studies on platelet-depleted mice and mice restored with activated platelets, which showed that eosinophil recruitment after lung antigen challenge required activated platelets in a P-selectin-dependent manner (Pitchford et al., 2005).

**Table 2:** **Evidence for eosinophil integrin activating stimuli *in vivo***.

Location	Integrin	Activation state	Activating stimuli	Evidence and reference
Blood	β_1_ integrins	Intermediate	P-selectin	Eosinophil-bound P-selectin correlates with N29 and 8E3 reactivities (Johansson and Mosher, 2011; Johansson et al., 2012), platelet-surface P-selectin correlates with N29 reactivity (Johansson et al., 2012)
	β_2_ integrins	Intermediate	IL-5 (low concentrations)	Anti-IL-5 decreases KIM-127 reactivity (Johansson et al., 2013a)
Airway	β_1_ integrins	High	Unknown	Johansson et al. (2013a)
	β_2_ integrins	High	Unknown (IL-5 may play a role but not required)	BAL fluid IL-5 concentration correlates with mAb24 reactivity (Johansson et al., 2008), but anti-IL-5 does not decrease mAb24 and CBRM1/5 reactivities (Johansson et al., 2013a)

*BAL, bronchoalveolar lavage; IL-5, interleukin-5*.

As alluded to above, IL-5 at concentrations ≥1 ng/ml (∼40 pM) induces blood eosinophil reactivity with CBRM1/5 and mAb24, and adhesion to α_M_β_2_ ligands *in vitro* (Zhu et al., 1999; Barthel et al., 2006a,b; Johansson and Mosher, 2011; Johansson et al., 2013b). *In vivo*, the KIM-127 signal of blood eosinophils was decreased in subjects with non-severe asthma after administration of anti-IL-5 mepolizumab (Table 2) (Johansson et al., 2013a), indicating that the intermediate conformation of β_2_ integrins on blood eosinophils is the result of exposure to IL-5 in the bone marrow and/or circulation. This IL-5 concentration is presumably low, since it does not result in significant expression of the mAb24 and CBRM1/5 epitopes in fully activated α_M_β_2_; and the IL-5 concentration in the blood of subjects with asthma is only 1–10 pg/ml (0.04–0.4 pM) (Mastalerz et al., 2001; Joseph et al., 2004; Johnsson et al., 2011). Thus, KIM-127 reactivity may be a read-out of tonic *in vivo* IL-5 activity and may predict responsiveness to anti-IL-5.

As for airway eosinophils, we do not know what factor is responsible for their high-activity β_1_ integrin state. We speculate that it may be the result of the eosinophils having undergone arrest, transendothelial migration, and encounters with adhesive ligands. Current thinking in the leukocyte integrin field includes the possibility that outside-in signaling following ligand-binding by integrin in the intermediate conformation brings about the final stage of activation to the high-activity conformation (Evans et al., 2009; Hogg et al., 2011). Thus, for eosinophil α_4_β_1_, interaction with VCAM-1 during arrest and transmigration may lead to higher activation, as detected on airway eosinophils.

In our first segmental antigen challenge study, mAb24 reactivity of BAL eosinophils correlated with IL-5 concentration in BAL fluid (Table 2) (Johansson et al., 2008), indicating that IL-5 can be an *in vivo* stimulus for the high-activation state of airway eosinophil β_2_ integrins. The concentration of IL-5 in airway lining fluid *in vivo* is 0.1–100 ng/ml (Teran et al., 1999; Kelly et al., 2003; Johansson et al., 2008), based on the estimated 100-fold dilution during the recovery of BAL (Rennard et al., 1986; Johansson et al., 2008). However, in contrast to blood eosinophil reactivity with KIM-127; BAL eosinophil reactivities with KIM-127, mAb24, and CBRM1/5 did not decrease after anti-IL-5 administration (Table 2) (Johansson et al., 2013a). Thus, the high-activity α_M_β_2_ state of airway eosinophils does not appear to require IL-5. Presumably, IL-3 and/or GM-CSF, which can induce adhesion to an α_M_β_2_ ligand *in vitro* (Johansson et al., 2013b) and which are estimated to be present at up to 10 ng/ml in airway lining fluid (Woolley et al., 1995; Evans et al., 1996; Jarjour et al., 1997; Johansson et al., 2008), may, possibly together with other factors, be responsible for the highly activated α_M_β_2_ on airway eosinophils *in vivo*.

In conclusion, P-selectin or IL-5 appears responsible for the partial activation of α_4_β_1_ or α_M_β_2_, respectively, on blood eosinophils. These data demonstrate that different integrins on eosinophils are activated by distinct stimuli and presumably by distinct signaling pathways, which is in accordance with a recent statement that “it is clear that mechanisms of activation are not generic for all integrins” (Margadant et al., 2011). In contrast, it is uncertain which factor or combinations of factors bring about the high-activation state of α_4_β_1_ and α_M_β_2_ on airway eosinophils. IL-3, GM-CSF, and/or other agents, possibly in synergy, may be responsible for the high-activity α_M_β_2_. IL-5 may play a minor, but dispensable, role. Interactions with counter-receptors or ligands during arrest, and transendothelial and continued migration may be responsible for high-activity α_4_β_1_ and may also contribute to high-activity α_M_β_2_.

## Correlations with Eosinophil Recruitment and Aspects of Asthma

Although it was known that purified blood eosinophils adhere “constitutively” to VCAM-1 *in vitro* (Walsh et al., 1991; Weller et al., 1991; Schleimer et al., 1992; Johansson et al., 2004; Barthel et al., 2006a,b), the possibility existed that the activation state of α_4_β_1_ *in vivo* would be variable and a determinant of eosinophil arrest on activated endothelium and consequently recruitment to the airway. We first tested this possibility in a double-blind placebo-controlled, two-period crossover inhaled corticosteroid (ICS) withdrawal study in subjects with non-severe asthma. N29 reactivity of blood eosinophils was found to correlate inversely with forced expiratory volume in 1 s (FEV_1_, as percentage of baseline) after ICS withdrawal or across all visits during the whole study (Table 3) (Johansson et al., 2006). Receiver-operator characteristic (ROC) curve analysis demonstrated that the N29 signal predicted decrease in FEV_1_ (Table 3) (Johansson et al., 2006). N29 correlated better with FEV_1_ and performed better in ROC analysis than did the established asthma biomarkers sputum eosinophil percentage and fraction of exhaled nitric oxide (FENO) (Johansson et al., 2006). Further, N29 correlated with FENO, believed to reflect airway inflammation, after ICS withdrawal (Table 3) (Johansson et al., 2006). These findings indicated that greater β_1_ activation (i.e., higher proportion of β_1_ integrins in the intermediate conformation vs. in the inactive conformation) on circulating eosinophils is associated with a higher degree of airway inflammation and decreased pulmonary function in non-severe asthma, presumably because subjects with a more activated α_4_β_1_ would have a higher degree of eosinophil arrest and recruitment to the airway.

**Table 3:** **Correlations between eosinophil integrin activation states and eosinophil recruitment or aspects of asthma**.

Location	Integrin: activation state (mAb)	Correlation	Reference
Blood	β_1_ Integrins: intermediate (N29)	Inverse with FEV_1_ (% of baseline) after or during ICS withdrawal in non-severe asthma, predicts decrease in FEV_1_ in receiver-operator characteristic (ROC) curve analysis	Johansson et al. (2006)
		FENO after ICS withdrawal in non-severe asthma	Johansson et al. (2006)
		Inverse with FEV_1_/FVC in younger subjects with non-severe asthma	Johansson et al. (2012)
		Inverse with FEV_1_/FVC in cluster 1 (mild atopic asthma)	Johansson et al. (2011)
	(48 h after segmental antigen challenge)	Response phenotype (increased vs. 0 h in dual, not single, responders)	Johansson et al. (2008)
	(48 h after segmental antigen challenge)	Late-phase fall in% FEV_1_ after whole-lung antigen challenge	Johansson et al. (2008)
	β_2_ Integrins: intermediate (KIM-127) (before but not after anti-IL-5)	% BAL eosinophils	Johansson et al. (2013a)
Airway	β_1_ Integrins: intermediate-high (N29)	% BAL eosinophils	Johansson et al. (2008)
		Response phenotype (higher in dual than in single responders)	Johansson et al. (2008)
	β_2_ Integrins: high (mAb24)	% BAL eosinophils	Johansson et al. (2008)
		Late-phase fall in% FEV_1_ after whole-lung antigen challenge	Johansson et al. (2008)

*BAL, bronchoalveolar lavage; FEV_1_, forced expiratory volume in 1 s; FVC, forced vital capacity; ICS, inhaled corticosteroid*.

To test the hypothesis that blood eosinophil β_1_ activation is clinically relevant in asthma in another model, we used segmental lung antigen challenge of subjects with non-severe allergic asthma. Blood eosinophil N29 reactivity 48 h after segmental challenge correlated with the late-phase fall in FEV_1_ 3–8 h after the whole-lung antigen challenge performed during screening (Table 3) (Johansson et al., 2008). N29 signal of airway eosinophils obtained 48 h after segmental challenge correlated with eosinophil percentage in BAL and was higher in dual than in single responders (Table 3) (Johansson et al., 2008). These results confirmed that degree of β_1_ activation on blood eosinophils is associated with pulmonary function in non-severe asthma and also showed that the β_1_ activation that occurs on airway eosinophils is associated with eosinophil recruitment. Unfortunately, blood during the screening whole-lung antigen challenge part of this study was not analyzed by flow cytometry. It would be interesting to investigate more directly correlations between integrin activation during whole-lung antigen challenge and measures of eosinophil recruitment, airway inflammation, and the late-phase response, by comparing flow cytometry data and clinical parameters at different time points after whole-lung challenge.

The ICS withdrawal and antigen challenge studies were on subjects with non-severe asthma who were young adults (mean 21 years old, only one of 27 subjects older than 30) (Johansson et al., 2008). To extend the study on the relationships between β_1_ integrin activation on blood eosinophils and pulmonary function to subjects with disease of varying severity and a greater age range, we performed an observational study in a population comprising both non-severe and severe asthma subjects with a higher mean age (34 for those with severe and 29 for those with non-severe asthma). As there was no baseline in this observational study, we examined correlations with FEV_1_ corrected for forced vital capacity (FVC). The correlation between N29 reactivity of blood eosinophils with FEV_1_/FVC did not reach significance in the whole asthma study population (Johansson et al., 2012). However, when subjects were stratified by severity and age, N29 correlated significantly with FEV_1_/FVC in those with non-severe asthma under 30 years of age (Table 3) (Johansson et al., 2012). The subjects in this study belonged to a population that had been classified using unsupervised hierarchical cluster analysis, resulting in five asthma phenotype groups (Moore et al., 2010). N29 correlated best and significantly with FEV_1_/FVC in cluster 1 (Johansson et al., 2011), which consists of subjects with mild atopic asthma (Moore et al., 2010).

Taking ICS withdrawal, antigen challenge, and the severe asthma studies together, it appears that greater β_1_ integrin activation on blood eosinophils is associated with decreased pulmonary function in subjects with non-severe asthma who are relatively young, but that this association breaks down in severe asthma or in older subjects. A possible explanation for the lack of association in severe asthma is high degree of ongoing extravasation of eosinophils with the most activated α_4_β_1_, as discussed above. An additional possible explanation is that a proportion of subjects with severe asthma do not have a predominantly eosinophilic airway inflammatory phenotype but rather a mixed eosinophilic-neutrophilic or a neutrophilic phenotype (Hastie et al., 2010; Wenzel, 2012), which may contribute to the weakening of the association between eosinophil activation and lung function. Further, it is unclear why the association does not hold up in older subjects. Perhaps pulmonary function becomes less associated with eosinophil activation and recruitment as the disease evolves with time and increased age. Such a situation may be related to the observation that neutrophilic inflammation in asthma is more frequent in older adults (Wang et al., 2011; Agache et al., 2012).

Regarding β_2_ integrins, the first antigen challenge study demonstrated that reactivity of BAL eosinophil mAb24 reactivity, like their N29 reactivity, correlated with percentage of eosinophils in BAL (Table 3) (Johansson et al., 2008). Further, the mAb24 signal of BAL eosinophils correlated with the magnitude of the late-phase response after whole-lung antigen challenge (Table 3) (Johansson et al., 2008). In the anti-IL-5 study, blood eosinophil reactivity with KIM-127 at the time of segmental challenge (before but not after anti-IL-5 administration) correlated with BAL eosinophil percentage 48 h later (Table 3) (Johansson et al., 2013a). Unfortunately, KIM-127 was not assayed in the earlier studies, so the repeatability of this observation is not known. However, these data indicate that the degree of (intermediate) β_2_ integrin activation on blood eosinophils and degree of high β_2_ activation achieved on airway eosinophils are also associated with eosinophil recruitment. These correlations offer support for the view that activation of β_2_ integrins, presumably α_M_β_2_, complements activation of α_4_β_1_, in mediating eosinophil arrest and movement to the airway.

## Conclusions and Perspectives

We review information on the activation state of integrins on blood and airway eosinophils, the likely *in vivo* activators of eosinophil integrins, and correlations between eosinophil integrin activation and measurements of eosinophil recruitment, airway inflammation, and pulmonary function in asthma. We concentrate on studies in humans and include results of studies done on mice.

The information indicates that a proportion of α_4_β_1_ integrin on circulating blood eosinophils is in the intermediate-activity conformation as a result of stimulation of eosinophils by P-selectin present on the surface of activated platelets binding to eosinophil PSGL-1. The proportion is variable among individuals, and thus α_4_β_1_ activation, by conferring variable capacity to arrest on VCAM-1 on activated endothelium in inflamed lung, seems to be a biomarker of disease activity in younger individuals with mild asthma. A proportion of α_M_β_2_ on blood eosinophils is also in the intermediate-activity conformation, presumably as a result of exposure to IL-5. Partially activated α_M_β_2_ likely assists in eosinophil arrest on and extravasation from inflamed vessels, and dampening of α_M_β_2_ activation may contribute to the therapeutic efficacy of anti-IL-5.

The classical multistep paradigm for extravasation of leukocytes (Springer, 1994), which has been applied to eosinophils (Rosenberg et al., 2007), depicts circulating leukocytes as having inactive integrins that become activated when rolling cells are exposed to chemokines associated with the surface of activated endothelium. The paradigm needs to be modified to include *in vivo* “pre-activation” or “priming” (Koenderman et al., 1996) mediated by P-selectin and IL-5 causing eosinophils to display integrins in partially activated conformations and leading to more efficient arrest. The modified paradigm is in accord with the conclusion that “increasing evidence suggests that subsets of circulating leukocytes express a fraction of their integrins in pre-formed intermediate-affinity states” that arrest or slow down rolling leukocytes (Alon and Dustin, 2007).

Airway eosinophils obtained by BAL 48 h after segmental antigen challenge have α_4_β_1_ and α_M_β_2_ in high-activity conformations, likely as a result of encounters with multiple factors in inflamed lung including, in the case of high-activity α_M_β_2_, exposure to IL-3, GM-CSF, and other soluble agents, and/or interaction with ligands such as VCAM-1 (for both integrins) and periostin (for α_M_β_2_). It is difficult to say much more without knowing the histories of the sampled cells as the cells responded to the challenge. Interestingly, degree of activation of both β_1_ and β_2_ integrins on airway eosinophils correlates with number of eosinophils found in BAL.

Observations on human blood eosinophils and genetically manipulated mice indicate that partially activated α_4_β_1_ and α_M_β_2_ on blood eosinophils cooperate in eosinophil arrest in vessels of inflamed bronchi and movement of eosinophils into lung tissue. α_4_β_1_ may dominate in arrest on VCAM-1, and α_M_β_2_ may dominate at the later stage by mediating extravasation and migration in the ECM. It should be stressed that some of the observations in humans are correlational in nature and have not yet been replicated by others, and a number of predictions based on the observations have not been tested. For instance, identification of the vascular beds in which partial eosinophil integrin activation takes place will firm up the observations. These need not to be the beds into which eosinophils ultimately extravasate. We have suggested that eosinophils may encounter activated platelets and P-selectin (Johansson and Mosher, 2011; Johansson et al., 2012) in the pulmonary circulation, which is sampled many times per hour by all blood cells, and thus the eosinophils are primed to extravasate when coursing through the systemic circulation of the inflamed bronchus, which would happen much less often (Johansson et al., 2008). It is not known whether the diminution of β_2_ integrin activation by anti-IL-5 is due to neutralization of IL-5 present in low concentration in the general circulation or at higher concentrations in certain vascular beds. Longitudinal studies are needed to relate changes in integrin activation to the natural history of asthma and thus supplement correlations done on values obtained at a single time point. Such studies may give insight into why β_1_ integrin activation on blood eosinophils correlates with pulmonary function in younger adults with non-severe asthma but not in patients with severe disease or older patients. Murine experiments are needed to assess the arrest and extravasation of eosinophils in intact blood vessels imaged in real time and relate eosinophil behavior to integrin activation state. Similar experiments have provided important information about the behavior of platelets and neutrophils in injured or inflamed vessels. Finally, more needs to be known about roles of eosinophil integrins in modulating the movement and behavior of eosinophils in lung tissues. Such studies may reveal important roles for the five eosinophil integrins that are largely ignored in this review.

## Conflict of Interest Statement

The authors declare that the research was conducted in the absence of any commercial or financial relationships that could be construed as a potential conflict of interest.
